# Activity of Antifungal Organobismuth(III) Compounds Derived from Alkyl Aryl Ketones against *S. cerevisiae*: Comparison with a Heterocyclic Bismuth Scaffold Consisting of a Diphenyl Sulfone

**DOI:** 10.3390/molecules190811077

**Published:** 2014-07-29

**Authors:** Toshihiro Murafuji, Mai Tomura, Katsuya Ishiguro, Isamu Miyakawa

**Affiliations:** 1 Graduate School of Medicine, Yamaguchi University, Yamaguchi 753-8512, Japan; 2 Graduate School of Science and Engineering, Yamaguchi University, Yamaguchi 753-8512, Japan

**Keywords:** bismuth, Lewis acidity, hypervalent, *S. cerevisiae*, antifungal activity, acetophenone

## Abstract

A series of hypervalent organobismuth(III) compounds derived from alkyl aryl ketones [XBi(5-R'C_6_H_3_-2-COR)(Ar)] was synthesized to investigate the effect of the compounds’ structural features on their antifungal activity against the yeast *Saccharomyces cerevisiae*. In contrast to bismuth heterocycles [XBi(5-RC_6_H_3_-2-SO_2_C_6_H_4_-1'-)] derived from diphenyl sulfones, a systematic quantitative structure-activity relationship study was possible. The activity depended on the Ar group and increased for heavier X atoms, whereas lengthening the alkyl chain (R) or introducing a substituent (R') reduced the activity. IBi(C_6_H_4_-2-COCH_3_)(4-FC_6_H_4_) was the most active. Its activity was superior to that of the related acyclic analogues ClBi[C_6_H_4_-2-CH_2_N(CH_3_)_2_](Ar) and ClBi(C_6_H_4_-2-SO_2_ *tert*-Bu)(Ar) and also comparable to that of heterocyclic ClBi(C_6_H_4_-2-SO_2_C_6_H_4_-1'-), which was the most active compound in our previous studies. Density function theory calculations suggested that hypervalent bismuthanes undergo nucleophilic addition with a biomolecule at the bismuth atom to give an intermediate ate complex. For higher antifungal activity, adjusting the lipophilicity-hydrophilicity balance, modeling the three-dimensional molecular structure around the bismuth atom, and stabilizing the ate complex appear to be more important than tuning the Lewis acidity at the bismuth atom.

## 1. Introduction

**Figure 1:**
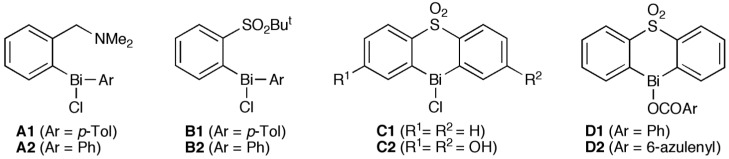
Molecular structure of hypervalent organobismuth compounds.

Biologically active bismuth compounds are the subject of considerable interest, although there have been only a few biological studies of organobismuth compounds [1,2,3,4,5,6]. This is in contrast to their growing use in synthetic reactions [7,8,9]. We recently reported the antifungal activity of hypervalent organobismuth(III) and (V) compounds against the yeast *Saccharomyces cerevisiae* [10,11,12]. The Lewis acidity at the bismuth center was essential for the activity. Compounds **A**–**D** are hypervalent organobismuth(III) derivatives, which are generally expressed by a “10-Bi-4” notation based on the number of total electrons in the outermost shell and bonds to the relevant heteroatom center [13,14] (Figure 1). Compounds **A** and **B** showed moderate activity but **C1** was much more active [10] (Table 1). The higher activity of **C1** can be attributed to its structural characteristics as well as the biopharmaceutical properties. Its lower ClogP value (1.18) indicates lower lipophilicity, the lack of bulky alkyl substituents on the sulfonyl group reduces steric crowding around the tetracoordinate bismuth atom, and weaker intramolecular coordination of the sulfonyl substituent arises from the longer intramolecular Bi–O atomic distance [2.775(6) Å] [10] because of the rigid heterocyclic structure and the highly coordinated geometry of the bismuth atom.

**Table 1:** Antifungal assay for bismuthanes.

Compound	Inhibition Zone (mm)	ClogP	Compound	Inhibition Zone (mm)	ClogP
**A1**	13	3.52	**6a**	8	2.54
**A2**	12	3.02	**6b**	12	3.12
**B1**	8	3.28	**6c**	16	2.63
**B2**	12	2.78	**6d**	17	2.77
**C1**	18	1.18	**6e**	15	3.34
****C2****	8	0.81	**6f**	0	4.12
**D1**	17	3.49	**7a**	9	2.54
**D2**	12	4.66	**7b**	15	3.12
			**7c**	17	2.63
**4b**	0	5.81	**7d**	19	2.77
**4c**	0	4.82	**7e**	14	3.34
**4d**	0	5.10	**7f**	0	4.12
**4e**	0	6.24	**9a**	10	3.62
**5a**	8	2.54	**9b**	7	4.55
**5b**	13	3.12	**9c**	10	3.34
**5c**	15	2.63	**11a**	9	4.18
**5d**	16	2.77	**11b**	8	4.71
**5e**	14	3.34	**11c**	10	3.96
**5f**	0	4.12	**Nystatin**	30	

In particular, the antifungal activity of monosubstituted **C** (R^2^ = H) decreases as ClogP increases, which in turn depends on substituent R^1^ [11]. On the basis of this relationship, we synthesized **C2**, which has hydrophilic substituents, but its activity was less than that of parent **C1** [11]. Yet, compared with **C1**, bismuth carboxylates **D** showed moderate to high activity despite their much higher lipophilicity. To explain this, we proposed that in the yeast cell the bismuth-carboxylate or bismuth-halogen bonds are cleaved into a cationic bismuth scaffold and a carboxylate or halide anion, and that the bismuth scaffold plays an important role in the inhibition activity [12]. Stable cationic organobismuth complexes have been isolated and characterized [15,16]. These findings indicate that antifungal activity is not just affected by the hydrophilicity or lipophilicity.

**Scheme 1:**
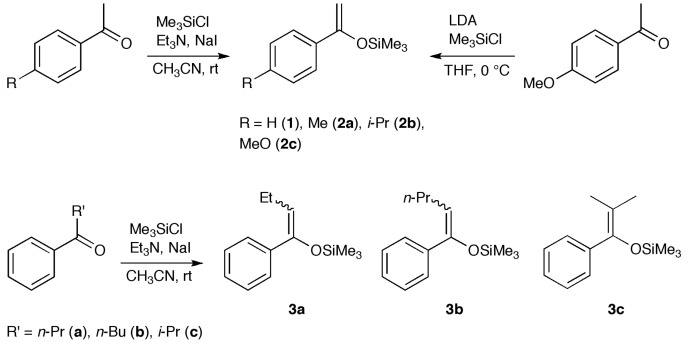
Synthesis of silyl enol ethers.

**Scheme 2:**
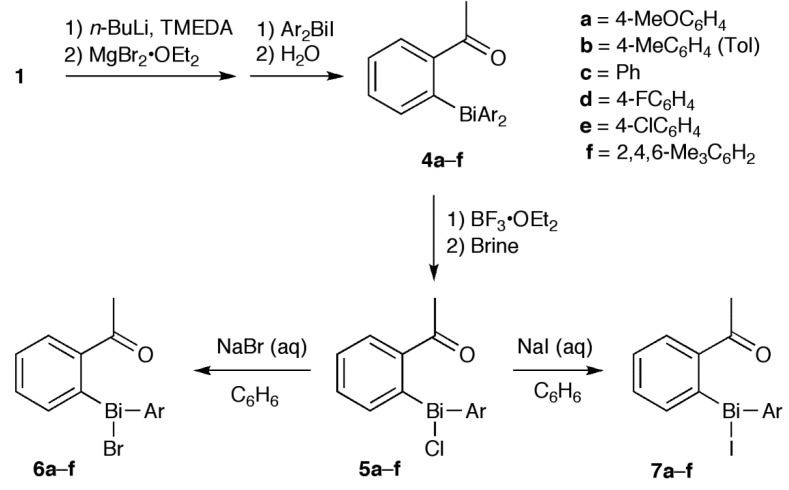
Synthesis of bismuthanes **4** and their transformation.

**Scheme 3:**
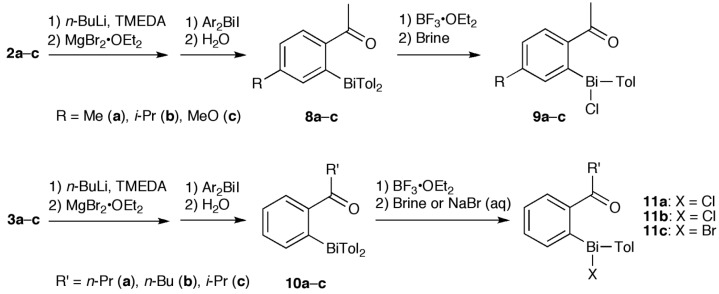
Synthesis of bismuthanes **8** and **10** and their transformation.

To design bismuthanes that are more active than **C1**, it is important to understand how their structural features affect their antifungal activity. Toward this end, we studied a series of halobismuthanes (**5**–**7**, **9** and **11**) derived from alkyl aryl ketone (Scheme 1, Scheme 2 and Scheme 3). Unlike the diphenyl sulfone structure of the heterocyclic bismuth scaffold **C**, the structures in this series could be systematically modified by changing the alkyl and aryl groups of the ketone scaffold as well as the aryl and halo groups attached to the bismuth atom. Furthermore, the various biological activities of acetophenone derivatives were attractive from the viewpoint of bioavailability [17,18,19]. Through this study, we aimed to better elucidate the effect of structural characteristics on the antifungal activity and also to design a halobismuthane (**7d**) that was comparable to **C1** in activity.

## 2. Results and Discussion

### 2.1. Synthesis

Silyl enol ethers **1**–**3** were prepared from the parent ketones [20,21] and used as starting materials (Scheme 1). We have previously reported the synthesis of **4a**–**c** and **4e**, their corresponding bromides **6**, and **5b** and **7b**, but the yield of parent compound **4** is very low [22] because the lithiated silyl enol ethers [23] and chlorobismuthanes are reactive. Hence, **4** and its homologues **8** and **10** were synthesized by modifying our previous method (Scheme 2 and Scheme 3). The reactive lithiated silyl enol ethers and chlorobismuthanes (Ar_2_BiCl) were replaced by the less reactive magnesiated derivatives and iodobismuthanes (Ar_2_BiI), respectively. This made the synthesis of triarylbismuthanes **4**, **8**, and **10** more reliable. The triarylbismuthanes were converted into the corresponding halobismuthanes **5**–**7**, **9**, and **11** by treatment with boron trifluoride diethyl ether complex and subsequent halogenation. Because **4a**, **4f**, and **10** decomposed during purification by column chromatography, they were isolated as the corresponding halobismuthanes. Compound **10c** was isolated as bromobismuthane **11c** because the corresponding chlorobismuthane was unstable. In the ^1^H-NMR spectra of the halobismuthanes, the *ortho* proton adjacent to the bismuth atom in the aryl ketone scaffold underwent anisotropic deshielding because of its close proximity to the electronegative chlorine, bromine or iodine atom attached to the bismuth center [24]. The *ortho* proton signal for **5b**, **6b**, and **7b** appeared at *δ* 9.06, 9.21 and 9.42 ppm, respectively. Furthermore, compared with parent bismuthane **4b**, the carbonyl carbon signal in the ^13^C-NMR spectra of **5b**, **6b**, and **7b** was shifted downfield by 6–8 ppm. The carbonyl stretching vibration in the IR spectrum of the halobismuthanes also showed a shift to a lower frequency by 40 cm^−1^. These observations indicate that the carbonyl oxygen atom was coordinated to the bismuth atom to form a hypervalent C=O•••Bi–Cl, C=O•••Bi–Br, or C=O•••Bi–I bonds. Hypervalent bond formation in **6b** has been confirmed by X-ray crystallography [22]. Halobismuthanes **5**–**7**, **9**, and **11** were stable in water and DMSO, indicating that the hypervalent bond formation suppresses hydrolysis and redistribution reactions of these compounds.

### 2.2. Growth Inhibition Tests against S. cerevisiae

**Figure 2:**
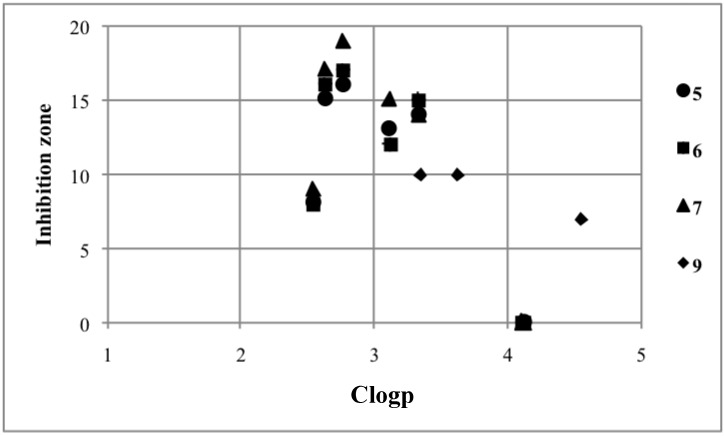
Plot of the antifungal assay for bismuthanes **5**–**7** and **9**.

Initially, we tested the inhibition activity of **4**–**7** ( Experimental Subsection 3.6, Table 1, and Figure 2). Triarylbismuthanes **4b**–**e** were inactive, but halobismuthanes **5**–**7** showed moderate to high antifungal activity. This is consistent with our previous finding that the Lewis acidic bismuth center is the active site [10,11,12]. Iodobismuthanes **7** showed higher activity than bromobismuthanes **6**, which were slightly more active than chlorobismuthanes **5**. This suggests that the activity increases with decreasing the electronegativity of the halo substituent. It should be noted that halobismuthanes **5d**, **6d**, and **7d** bearing a *p*-fluorophenyl group showed the highest activity among each compound’s halogen homologues **a**–**f**. Although **5a**, **6a**, and **7a** bearing a *p*-methoxyphenyl group were the most hydrophilic among each compound’s homologues, they were not very active because their solubility was reduced by the aryl group [25]. Halobismuthanes **5f**, **6f**, and **7f** bearing a mesityl group were inactive. Next, we determined the inhibition activity of **9a**–**c**, which were prepared from *p*-substituted acetophenones. The activity of **9a**–**c** was reduced by the *p*-substituent. This trend is similar to that observed in the quantitative structure-activity relationship study of **C**. In view of the higher activity of halobismuthanes **5d**, **6d**, and **7d**, an attempt to synthesize **9** bearing a *p*-fluoro substituent in the acetophenone scaffold failed: It was difficult to control the lithiation of the corresponding silyl enol ether because the electronegativity of the fluoro substituent increased the reactivity of the ether toward the lithiation. We replaced the methyl substituent of the acetyl group in **5b** with various alkyl substituents to obtain **11a**–**c**, but they were less active. Halobismuthane **7d** showed the highest activity of the compounds in this study, and its activity was also comparable to that of **C1**. Although the activity of **7d** was not as high as the standard antifungal drug, nystatin, this finding demonstrates the high antifungal activity of organobismuth compounds. A certain organobismuth compound has been reported to exhibit selective cytotoxicity to vascular endothelial cells, which may be applicable in antivascular cancer therapy [26]. However, it remains to be determined whether **7d** and its related compounds exhibit specific cytotoxicity against fungi.

### 2.3. Structure-Activity Relationship

Based on this study, the structure-activity relationship of the hypervalent bismuthanes may be summarized as follows. Firstly, steric crowding around the tetracoordinate bismuth atom substantially lowers antifungal activity. The lack of activity of **5f**, **6f**, and **7f** was attributed to the bulky mesityl group, which possibly blocks the interaction of the bismuth atom with the biomolecules in the yeast. The long chain or branched RCO group in **11a**–**c** may also play a similar role, as suggested by **A** and **B** bearing Me_2_N and *tert*-BuSO_2_ groups, respectively. Secondly, the hydrophilicity of the hypervalent bismuthanes is not always proportional to their antifungal activity, as was observed in **5a**, **6a**, and **7a**, and **C2**. In particular, **7d** is less hydrophilic (ClogP = 2.77) than **C1** (ClogP = 1.18) but comparably more active. This suggests that the relationship in homologous series **C** between activity and ClogP does not apply to **7d**, which contains acetophenone, and is completely different from diphenyl sulfone in terms of structure and properties. Thirdly, the heterocyclic structure in **C1** is not essential for high antifungal activity. Fourthly, introducing a fluoro substituent at an appropriate position in the hypervalent bismuthanes enhances the antifungal activity. The enhancement of biological activity by fluoro substituents is well known in drugs and pesticides [27,28,29]. Fifthly, the Lewis acidity at the bismuth atom, although essential, does not correspond directly to the antifungal activity. Thus, **5a**, **5f**, **6b**, and **7d** showed a wide variation in activity depending on the aryl and halo groups (Table 1), although the Lewis acidity of the bismuth atoms of these compounds were similar as assessed by the carbonyl carbon signals (*δ* ppm) and the carbonyl stretching bands (*ν* cm^−1^) in the ^13^C-NMR and IR spectra, respectively (**5a**, 209.2, 1620; **5f**, 208.1, 1630; **6b**, 208.7, 1625; and **7d**, 207.7, 1630). Therefore, we compared the Lewis acidity at the tetracoordinate bismuth atom between different scaffolds by using the intramolecular Bi–O and Bi–N distances and examined their relationship to the antifungal activity. The length of the metal-ligand bond has been used to quantify the Lewis acidity at the metal center [30,31]. We have previously determined the Bi–O or Bi–N intramolecular distance in **6b** [22], **A1** [32], **B1** [24], **C1** [10], **C2** [11], and **D2** [12] by X-ray crystallography. We expected that the metal-ligand interactions would decrease as the intramolecular distance is shortened because the metal-ligand interactions would reduce the Lewis acidity at the metal center [33]. Hence, the Lewis acidity in these compounds should be in the order **C1** [2.775(6) Å] > **D2** [2.754(2) Å] > **C2** [2.634(7) Å] > **B1** [2.592(5) Å] > **A1** [2.525(6) Å] > **6b** [2.519(7) Å]. However, the antifungal activity decreased in the order **C1** > **A1** > **D2**, **6b** > **B1**, **C2**, which did not correspond to the order of the intramolecular distance. Although hypervalent bond formation is known to enhance the Lewis acidity at the metal center [34,35,36,37,38], the enhancement of the Lewis acidity at the bismuth atom in these bismuthanes, even if it does occur, appears to be very slight because only one electron-accepting Bi–X bond is present.

### 2.4. Plausible Mechanism of Action for Hypervalent Bismuthanes

**Scheme 4:**
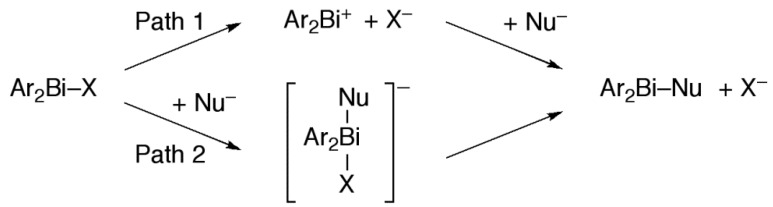
Plausible reaction pathway of the bismuthane.

We have proposed that C and D undergo cleavage of the Bi–Cl or Bi–O bond in the yeast cell to form a cationic bismuth scaffold, which plays an important role in the inhibition activity [12]. A plausible pathway for this reaction is shown as Path 1 in Scheme 4. Although we have not identified the biomolecules to which these bismuthanes bind, we expect that the Lewis acidic bismuth atom should have a high affinity for thiol groups [39]. Finally, we examined how the dissociation energy of the bismuth-halogen or bismuth-oxygen bond in Path 1 affects the antifungal activity. Using density functional theory [40], we calculated the dissociation energy (enthalpies) of these bonds in **A**–**D**, **5**–**7**, and **9** (Table 2, Path 1).

**Table 2:** Dissociation and association energy.

Ar_2_BiX	Inhibition Zone (mm)	Path 1 (kcal/mol)	Path 2 (kcal/mol)	Ar_2_BiX	Inhibition Zone (mm)	Path 1 (kcal/mol)	Path 2 (kcal/mol)
**A1**	13	12.82	–8.18	**6a**	8	17.93	–12.29
**A2**	12	13.12	–8.46	**6b**	12	18.38	–12.33
**B1**	8	19.33	–13.51	**6c**	16	18.73	–12.65
**B2**	12	19.66	–13.85	**6d**	17	19.06	–13.29
**C1**	18	25.22	–17.22	**6e**	15	19.37	–13.38
**C2**	8	24.75	–17.15	**6f**	0	18.15	–
**D1**	17	36.93	–10.09	**7a**	9	15.81	–15.18
**D2**	12	35.49	–10.97	**7b**	15	16.22	–15.13
**5a**	8	16.93	–12.08	**7c**	17	16.59	–15.43
**5b**	13	17.37	–12.00	**7d**	19	16.89	–16.00
**5c**	15	17.75	–12.33	**7e**	14	17.14	–16.26
**5d**	16	18.07	–13.02	**7f**	0	18.15	-
**5e**	14	18.36	–13.14	**9a**	10	16.83	–11.71
**5f**	0	18.15	-	**9c**	10	16.54	–11.32

**Figure 3:**
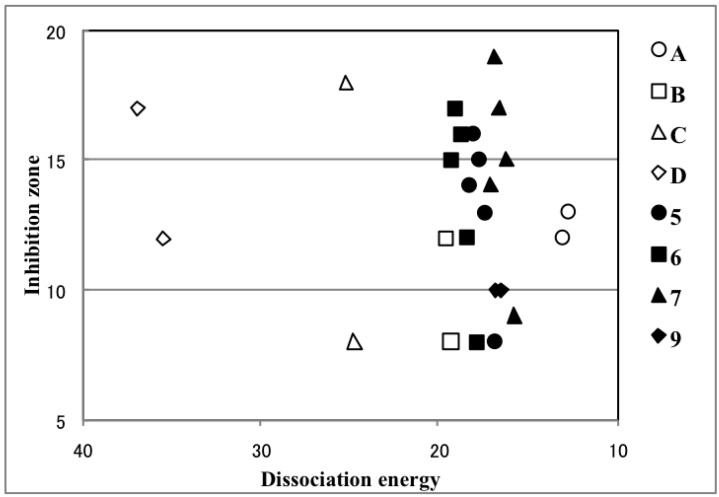
Plot of the dissociation energy against the antifungal activity.

**Figure 4:**
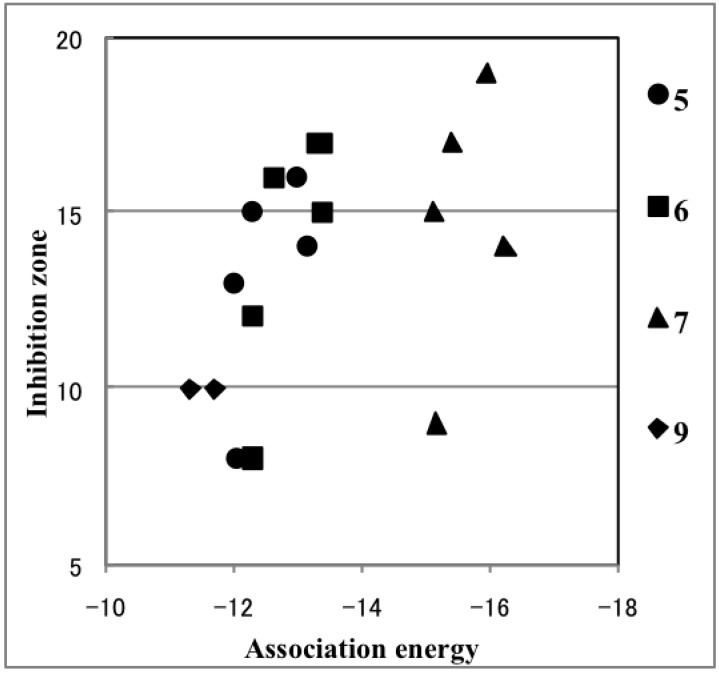
Plot of the association energy against the antifungal activity.

Contrary to our expectation, very active **C1** and **D1** had highly endothermic dissociation energy (25.22 and 36.93 kcal/mol, respectively), indicating that generating the bismuth cation requires high energy. Furthermore, the dissociation energy of moderately active **A1** was quite low owing to the stabilization of the bismuth cation by a strong coordination with the dimethylamino group. However, the most active compound, **7d**, was not as endothermic (16.89 kcal/mol), which is consistent with our proposed mechanism. Comparing the dissociation energy of **7a**, **7c**, and **7d**, and of **5a**, **5c**, and **5d**, revealed that the *p*-fluorophenyl group in **5d** and **7d** destabilizes the bismuth cation. No correlation was observed between dissociation energy and antifungal activity (Figure 3). Hence, we concluded that the bismuth cations are not generated from hypervalent bismuthanes in yeast cells. Alternatively, we propose that an intermediate ate complex [Ar_2_BiXNu]^−^ is formed by the bismuthanes undergoing addition at the bismuth atom with biomolecules such as hydrolases (Scheme 4, Path 2). After the release of X^−^, the resulting Ar_2_BiNu species is delivered to the organelle responsible for the antifungal activity via a further biological process. We calculated the association energy (enthalpies) by using methanethiolate anion as a model nucleophile (Nu^−^) (Table 2, Path 2). Among all the bismuthanes, **C1** and **7d** had some of the most exothermic association reaction, indicating that this path is preferable. Unlike Path 1, a weak but positive correlation between association energy and antifungal activity was observed (Figure 4). It should be stressed that in **5f**, **6f**, and **7f**, which contained a mesityl group, no minimum energy path connects the reactants and the product. This is in good agreement with their lack of activity. The activity of **7** was higher than that of **5** and **6**, although the activity of **C1** and its bromo and iodo analogues did not depend on the halo group [10]. This may be because the intramolecular Bi–O interaction of the carbonyl group is stronger than that of the sulfonyl group, which slightly enhances the Lewis acidity [34,35,36,37,38] at the bismuth atom and promotes the nucleophilic addition of Nu^−^. Such enhancement of the Lewis acidity has been observed in triorganotin halides bearing an intramolecular coordination with a carbonyl group [41].

## 3. Experimental Section

### 3.1. General Information

All reactions were carried out under argon unless otherwise noted. Hexane, diethyl ether and dichloromethane were distilled from calcium hydride before use. Melting points were determined on a Yanagimoto melting point apparatus without correction. ^1^H- and ^13^C-NMR spectra were recorded in CDCl_3_ or DMSO-*d_6_* on a Bruker Avance 400S spectrometer with TMS as an internal standard. IR spectra were obtained as KBr pellets on a Nicolet FT-IR Impact 410 spectrophotometer. Elemental analyses were performed on a Micro Corder JM10 apparatus (J-Science Lab. Co., Kyoto, Japan).

### 3.2. Synthesis of ***4***, ***8*** and ***10***

A typical example is exemplified by the synthesis of **4b**: To a stirred solution of TMEDA (1.36 mL, 9 mmol) in hexane (5 mL) was added dropwise at ice bath temperature *n*-butyllithium (9 mmol) in hexane followed by acetophenone trimethylsilyl enol ether **1** (576 mg, 3 mmol), and the mixture was stirred for 24 h at room temperature. In a separate flask, a mixture of bismuth(III) chloride (315.5 mg, 1 mmol) and tris(4-methylphenyl)bismuthane (964 mg, 2 mmol) was stirred in ether (10 mL) at room temperature for 1 h. To the suspension of chlorobis(4-methylphenyl)bismuthane (ca. 3 mmol) thus formed was added sodium iodide (450 mg, 3 mmol) and a few drops of 15-crown-5 ether and the resulting yellowish mixture was stirred for 3 h at room temperature. To a suspension of the lithium compound previously prepared was added at room temperature magnesium dibromide diethyl etherate (775 mg, 3 mmol) followed by, at −30 °C, a suspension of iodobis(4-methylphenyl)bismuthane (ca. 9 mmol), and the resulting mixture was stirred for 1 h, during which time the temperature was raised to ambient temperature. The reaction mixture was poured into brine (50 mL) and extracted with ethyl acetate (50 mL × 3). The combined extracts were concentrated to leave an oily residue, which was purified by chromatography (silica gel) using hexane-ethyl acetate (5:1) as the eluent to afford 4b in 30% yield (459 mg, 0.9 mmol). Because **4a**, **4f** and **10** underwent decomposition when purified by chromatography on silica gel, these compounds were converted into the corresponding halobismuthanes **5a**, **5f** and **11**, respectively, without isolation.

*(2-Acetylphenyl)bis(4-methylphenyl)bismuthane* (**4b**). Yield: 30%; mp 114–116 °C; ^1^H-NMR (400 MHz, CDCl_3_): *δ* 2.30 (6H, s), 2.62 (3H, s), 7.17 (4H, d, *J* = 7.8 Hz), 7.41 (1H, dt, *J* = 1.4, 7.6 Hz), 7.47 (1H, dt, *J* = 1.4, 7.6 Hz), 7.57 (4H, d, *J* = 7.8 Hz), 7.93 (1H, dd, *J* = 1.4, 7.6 Hz), 8.14 (1H, dd, *J* = 1.4, 7.6 Hz); ^13^C-NMR (100 MHz, CDCl_3_): *δ* 21.5, 27.2, 127.5, 131.2, 132.2, 135.0, 136.8, 137.7, 140.3, 141.5, 158.5, 159.8, 201.1. IR (KBr): ν = 1665, 1260, 790, 765, 600 and 480 cm^−1^. Anal. Calc. for C_22_H_21_BiO: C, 51.77; H, 4.15. Found: C, 51.70; H, 4.00%.

*(2-Acetylphenyl)diphenylbismuthane* (**4c**). Yield: 16%; mp 96–98 °C; ^1^H-NMR (400 MHz, CDCl_3_): *δ* 2.63 (3H, s), 7.29 (2H, t, *J* = 7.6 Hz), 7.36 (4H, t, *J* = 7.6 Hz), 7.42 (1H, dt, *J* = 1.2, 7.6 Hz), 7.49 (1H, dt, *J* = 1.4, 7.6 Hz), 7.69 (4H, d, *J* = 7.6 Hz), 7.91 (1H, dd, *J* = 1.2, 7.6 Hz), 8.16 (1H, dd, *J* = 1.2, 7.6 Hz). ^13^C-NMR (100 MHz, CDCl_3_): *δ* 27.2, 127.3, 127.6, 130.3, 132.3, 135.2, 137.7, 140.4, 141.5, 201.2; *ipso* carbon signals were too weak to be observed. IR (KBr): ν = 1660, 1260, 1090, 1000, 800, 760, 725, 700 and 600 cm^−1^. Anal. Calc. for C_20_H_17_BiO: C, 49.80; H, 3.55. Found: C, 49.60; H, 3.50%.

*(2-Acetylphenyl)bis(4-fluorophenyl)bismuthane* (**4d**). Yield: 11%; mp 147–149 °C; ^1^H-NMR (400 MHz, CDCl_3_): *δ* 2.64 (3H, s), 7.00–7.05 (4H, m), 7.46 (1H, dt, *J* = 1.6, 7.6 Hz), 7.52 (1H, dt, *J* = 1.6, 7.6 Hz), 7.58–7.62 (4H, m), 7.84 (1H, dd, *J* = 1.2, 7.2 Hz), 8.18 (1H, dd, *J* = 1.2, 7.6 Hz). ^13^C-NMR (100 MHz, CDCl_3_): *δ* 27.2, 117.7 (d, *J_CF_* = 19 Hz), 127.9, 132.5, 135.3, 139.3 (d, *J_CF_* = 6.0 Hz), 140.1, 141.3, 156.8 (br), 160.3 (br), 162.5 (d, *J_CF_* = 245 Hz), 201.4. IR (KBr): ν = 1660, 1570, 1480, 1210, 1160, 820, 770, 600 and 500 cm^−1^. Anal. Calc. for C_20_H_15_BiF_2_O: C, 46.35; H, 2.92. Found: C, 46.37; H, 2.88%.

*(2-Acetylphenyl)bis(4-chlorophenyl)bismuthane* (**4e**). Yield: 23%; mp 129–131 °C; ^1^H-NMR (400 MHz, CDCl_3_): *δ* 2.64 (3H, s), 7.30 (4H, d, *J* = 8.0 Hz), 7.46 (1H, dt, *J* = 1.2, 7.6 Hz), 7.52 (1H, dt, *J* = 1.2, 7.5 Hz), 7.57 (4H, d, *J* = 8.0 Hz), 7.84 (1H, dd, *J* = 1.2, 7.2 Hz), 8.19 (1H, dd, *J* = 1.2, 7.6 Hz). ^13^C-NMR (100 MHz, CDCl_3_): *δ* 27.2, 128.0, 130.6, 132.6, 133.7, 135.5, 139.0, 140.2, 141.3, 201.4; *ipso* carbon signals were too weak to be observed. IR (KBr): ν = 1660, 1260, 1090, 1000, 800, 765 and 710 cm^−1^. Anal. Calc. for C_20_H_15_BiCl_2_O: C, 43.58; H, 2.74. Found: C, 43.40; H, 2.70%.

*(2-Acetyl-4-methylphenyl)bis(4-methylphenyl)bismuthane* (**8a**). Yield: 18%; mp 148–150 °C; ^1^H-NMR (400 MHz, CDCl_3_): *δ* 2.22 (3H, s), 2.30 (6H, s), 2.59 (3H, s), 7.16 (4H, d, *J* = 7.6 Hz), 7.26 (1H, d, *J* = 7.6 Hz), 7.57 (4H, d, *J* = 7.6 Hz), 7.74 (1H, d, *J* = 1.3 Hz), 8.03 (1H, d, *J* = 7.6 Hz). ^13^C-NMR (100 MHz, CDCl_3_): *δ* 21.5, 21.6, 27.1, 128.2, 131.1, 132.2, 136.7, 137.7, 139.2, 141.03, 145.54, 158.48, 159.96, 200.6. IR (KBr): ν = 1660, 1580, 1290, 830, 790 and 480 cm^−1^. Anal. Calc. for C_23_H_23_BiO: C, 52.68; H, 4.42. Found: C, 52.32; H, 4.38%.

*(2-Acetyl-4-isopropylphenyl)bis(4-methylphenyl)bismuthane* (**8b**). Yield: 22%; mp 145–147 °C; ^1^H-NMR (400 MHz, CDCl_3_): *δ* 1.07 (6H, d, *J* = 7.2 Hz), 2.30 (6H, s), 2.60 (3H, s), 2.77 (1H, sep, *J* = 7.2 Hz), 7.16 (4H, d, *J* = 7.6 Hz), 7.30 (1H, dd, *J* = 1.6, 8.0 Hz), 7.58 (4H, d, *J* = 7.6 Hz), 7.78 (1H, d, *J* = 1.6 Hz), 8.06 (1H, d, *J* = 7.6 Hz). ^13^C-NMR (100 MHz, CDCl_3_): *δ*21.5, 23.4, 27.1, 34.0, 125.5, 131.0, 132.3, 136.7, 137.7, 139.5, 155.7, 158.6, 160.0, 200.5. IR (KBr): ν = 1650, 1580, 1350, 1190, 1050, 830, 790, 590 and 480 cm^−1^. Anal. Calc. for C_25_H_27_BiO: C, 54.35; H, 4.93. Found: C, 54.28; H, 4.84%.

*(2-Acetyl-4-methoxyphenyl)bis(4-methylphenyl)bismuthane* (**8c**). Yield: 8%; mp 135–137 °C; ^1^H-NMR (400 MHz, CDCl_3_): *δ*2.27 (3H, s), 2.32 (6H, s), 3.84 (3H, s), 7.02 (1H, d, *J* = 8.8 Hz), 7.20 (4H, d, *J* = 7.6 Hz), 7.58 (4H, d, *J* = 7.6 Hz), 7.99 (1H, dd, *J* = 2.0, 8.4 Hz), 8.15 (1H, d, *J* = 2.0 Hz). ^13^C-NMR (100 MHz, CDCl_3_): *δ* 21.4, 26.2, 55.8, 109.4, 130.6, 131.2, 133.1, 137.3, 137.5, 139.7, 143.0, 151.1, 165.6, 197.2. IR (KBr): ν = 1670, 1580, 1240, 1010, 800, 580 and 480 cm^−1^. Anal. Calc. for C_23_H_23_BiO_2_: C, 51.12; H, 4.29. Found: C, 50.88; H, 4.28%.

### 3.3. Synthesis of ***5a***, ***5f*** and ***11***

A typical example is exemplified by the synthesis of **5a**: An oily residue containing **4a** obtained by the concentration of the extracted organic layer was dissolved in dichloromethane (5 mL) and boron trifluoride etherate (3 mmol) was added to the solution at 0 °C until **4a** was completely consumed (checked by TLC). The mixture was diluted by the addition of brine (5 mL) and the organic layer was extracted with ethyl acetate (20 mL × 3). The combined extracts were concentrated to leave an oily residue, which was crystallized from MeOH to give **5a** in 18% yield.

*(2-Acetylphenyl)chloro(4-methoxylphenyl)bismuthane* (**5a**). Yield: 18%; mp 156–158 °C; ^1^H-NMR (400 MHz, CDCl_3_): *δ* 2.69 (3H, s), 3.73 (3H, s), 7.01 (2H, d, *J* = 8.8 Hz), 7.63 (1H, dt, *J* = 1.2, 8.8 Hz), 8.00 (1H, dt, *J* = 1.2, 8.8 Hz), 8.03 (2H, d, *J* = 8.8 Hz), 8.25 (1H, d, *J* = 8.0 Hz), 9.06 (1H, d, *J* = 6.8 Hz). ^13^C-NMR (100 MHz, CDCl_3_): *δ* 27.4, 55.0, 117.4, 128.3, 135.2, 138.0, 138.1, 138.3, 143.1, 159.5, 172.8, 185.3, 209.2. IR (KBr): ν = 1620, 1580, 1490, 1280, 1250, 810, 770 and 620 cm^−1^. Anal. Calc. for C_15_H_14_BiClO: C, 39.62; H, 3.10. Found: C, 39.42; H, 2.94%.

*(2-Acetylphenyl)chloro(2,4,6-trimethylphenyl)bismuthane* (**5f**). Yield: 13%; mp 172–174 °C; ^1^H-NMR (400 MHz, CDCl_3_): *δ*2.18 (3H, s), 2.46 (6H, s), 2.63 (3H, s), 7.12 (2H, s) 7.61 (1H, dt, *J* = 1.2, 7.6 Hz), 7.95 (1H, dt, *J* = 1.2, 7.6 Hz), 8.22 (1H, dd, *J* = 0.8, 7.6 Hz), 9.23 (1H, d, *J* = 7.6 Hz). ^13^C-NMR (100 MHz, CDCl_3_): *δ* 21.2, 26.1, 27.4, 128.1, 131.4, 134.9, 137.2, 138.6, 140.2, 144.1, 146.6, 182.4, 183.3, 208.1. IR (KBr): ν = 1630, 1290, 760 and 610 cm^−1^. Anal. Calc. for C_17_H_18_BiClO: C, 42.30; H, 3.76. Found: C, 42.10; H, 3.82%.

*(2-Butyrylphenyl)chloro(4-methylphenyl)bismuthane* (**11a**). Yield: 21%; mp 136–138 °C; ^1^H-NMR (400 MHz, CDCl_3_): *δ* 0.94 (3H, t, *J* = 7.6 Hz), 1.75 (2H, m), 2.25 (3H, s), 3.02 (2H, m), 7.24 (2H, d, *J* = 7.6 Hz), 7.69 (1H, t, *J* = 7.6 Hz), 7.86 (1H, t, *J* = 7.6 Hz), 8.07 (2H, d, *J* = 7.6 Hz), 8.24 (1H, d, *J* = 7.6 Hz), 9.41 (1H, d, *J* = 7.6 Hz). ^13^C-NMR (100 MHz, CDCl_3_): *δ* 13.7, 17.9, 21.5, 41.2, 128.1, 132.4, 134.5, 136.4, 137.9, 138.0, 138.4, 143.0, 178.0, 185.5, 211.6. IR (KBr): ν = 1620, 1220, 800, 760 and 480 cm^−1^. Anal. Calc. for C_17_H_18_BiClO: C, 42.30; H, 3.76. Found: C, 41.89; H, 3.69%.

*Chloro(2-valerylphenyl)(4-methylphenyl)bismuthane* (**11b**). Yield: 21%; mp 133–135 °C; ^1^H-NMR (400 MHz, CDCl_3_): *δ* 0.92 (3H, t, *J* = 7.6 Hz), 1.34 (2H, m), 1.70 (2H, m), 2.24 (3H, s), 3.05 (2H, m), 7.32 (2H, d, *J* = 7.6 Hz), 7.61 (1H, dt, *J* = 1.2, 7.6 Hz), 7.95 (1H, dt, *J* = 1.2, 7.6 Hz), 8.02 (2H, d, *J* = 7.6 Hz), 8.26 (1H, d, *J* = 7.6 Hz), 9.06 (1H, dd, *J* = 0.8, 7.6 Hz). ^13^C-NMR (100 MHz, CDCl_3_): *δ*13.8, 21.5, 22.3, 26.5, 39.1, 128.1, 132.4, 134.6, 136.4, 137.9, 138.1, 138.4, 143.0, 178.0, 185.5, 211.8. IR (KBr): ν = 1630, 1380, 1010, 800, 740 and 480 cm^−1^. Anal. Calc. for C_18_H_20_BiClO: C, 43.52; H, 4.06. Found: C, 43.88; H, 4.24%.

*Bromo(2-isobutyrylphenyl)(4-methylphenyl)bismuthane* (**11c**). Yield: 25%; mp 146–148 °C; ^1^H-NMR (400 MHz, DMSO-*d*_6_): *δ* 1.11 (6H, br-d, *J* = 4.1 Hz), 2.17 (3H, s), 3.89 (1H, sep, *J* = 6.8 Hz), 7.28 (2H, d, *J* = 7.6 Hz), 7.74 (1H, t, *J* = 7.6 Hz), 7.98 (2H, d, *J* = 7.6 Hz), 8.25 (1H, d, *J* = 7.6 Hz), 8.56 (1H, d, *J* = 7.6 Hz), 8.97 (1H, br-s). ^13^C-NMR (100 MHz, CDCl_3_): *δ* 19.1, 19.3, 21.5, 36.3, 128.2, 132.3, 134.3, 136.8, 137.9, 138.2, 140.9, 142.0, 174.4, 181.9, 215.1. IR (KBr): ν = 1620, 1260, 980, 770, 730 and 480 cm^−1^. Anal. Calc. for C_17_H_18_BiBrO: C, 38.73; H, 3.44. Found: C, 39.00; H, 3.63%.

### 3.4. Synthesis of ***5b**–**e*** and ***9***

A typical example is exemplified by the synthesis of **5b**: Compound **4b** (510 mg, 1 mmol) was dissolved in dichloromethane (5 mL) and boron trifluoride etherate (3 mmol) was added to the solution at 0 °C until **4b** was completely consumed (checked by TLC). The mixture was diluted by the addition of brine (5 mL) and the organic layer was extracted with ethyl acetate (20 mL × 3). The combined extracts were concentrated to leave an oily residue, which was crystallized from MeOH to give **5b** in 88% yield.

*(2-Acetylphenyl)chloro(4-methylphenyl)bismuthane* (**5b**). Yield: 88%; mp 164–166 °C; ^1^H-NMR (400 MHz, CDCl_3_): *δ* 2.25 (3H, s), 2.69 (3H, s), 7.33 (2H, d, *J* = 7.6 Hz), 7.62 (1H, dt, *J* = 1.2, 7.6 Hz), 7.99 (1H, dt, *J* = 1.2, 7.6 Hz), 8.03 (2H, d, *J* = 7.6 Hz), 8.24 (1H, dd, *J* = 0.8, 7.6 Hz), 9.06 (1H, dd, *J* = 0.8, 7.2 Hz). ^13^C-NMR (100 MHz, CDCl_3_): *δ* 21.5, 27.4, 128.2, 132.4, 135.2, 136.4, 138.1, 138.3, 143.1, 177.9, 185.2, 209.2; one *ipso* carbon signal was too weak to be observed. IR (KBr): ν = 1625, 1550, 1280, 800, 765, 620 and 480 cm^−1^. Anal. Calc. for C_15_H_14_BiClO: C, 39.62; H, 3.10. Found: C, 39.60; H, 3.10%.

*(2-Acetylphenyl)chloro(phenyl)bismuthane* (**5c**)*.* Yield: 82%; mp 152–154 °C; ^1^H-NMR (400 MHz, CDCl_3_): *δ* 2.69 (3H, s), 7.26 (1H, t, *J* = 7.8 Hz), 7.52 (2H, t, *J* = 8.0 Hz), 7.63 (1H, dt, *J* = 1.2, 7.6 Hz), 8.01 (1H, dt, *J* = 1.2, 7.6 Hz), 8.15 (2H, dd, *J* = 1.2, 8.0 Hz), 8.25 (1H, dd, *J* = 0.8, 7.2 Hz), 9.07 (1H, dd, *J* = 0.4, 8.0 Hz). ^13^C-NMR (100 MHz, CDCl_3_): *δ* 27.4, 128.2, 128.3, 131.6, 135.2, 136.4, 136.2, 138.3, 143.2, 181.0, 185.3, 209.2. IR (KBr): ν = 1620, 1220, 800, 740 and 480 cm^−1^. Anal. Calc. for C_14_H_12_BiClO: C, 38.16; H, 2.74. Found: C, 37.94; H, 2.80%.

*(2-Acetylphenyl)chloro(4-fluorophenyl)bismuthane* (**5d**). Yield: 79%; mp 130–132 °C; ^1^H-NMR (400 MHz, CDCl_3_): *δ* 2.70 (3H, s), 7.11–7.15 (2H, m), 7.64 (1H, dt, *J* = 1.2, 7.6 Hz), 8.01 (1H, dt, *J* = 1.2, 7.6 Hz), 8.07–8.12 (2H, m), 8.26 (1H, dd, *J* = 0.4, 7.6 Hz), 9.04 (1H, d, *J* = 7.2 Hz). ^13^C-NMR (100 MHz, CDCl_3_): *δ* 27.4, 118.8 (d, *J*_CF_ = 20 Hz), 128.5, 135.3, 138.2, 138.3, 138.5 (d, *J*_CF_ = 7.0 Hz), 143.1, 162.5 (d, *J*_CF_ = 246 Hz), 175.9, 185.1, 209.5. IR (KBr): ν = 1630, 1570, 1480, 1220, 1160, 830, 780, 610 and 500 cm^−1^. Anal. Calc. for C_14_H_11_BiClFO: C, 36.66; H, 2.42. Found: C, 36.73; H, 2.37%.

*(2-Acetylphenyl)chloro(4-chlorophenyl)bismuthane* (**5e**). Yield: 91%; mp 168–170 °C; ^1^H-NMR (400 MHz, CDCl_3_): *δ* 2.70 (6H, s), 7.43 (2H, d, *J* = 8.0 Hz), 7.64 (1H, dt, *J* = 1.2, 7.6 Hz), 8.02 (1H, dt, *J* = 1.2, 7.6 Hz), 8.08 (2H, d, *J* = 8.0 Hz), 8.26 (1H, dd, *J* = 0.8, 8.0 Hz), 9.05 (1H, dd, *J* = 0.8, 7.2 Hz). ^13^C-NMR (100 MHz, CDCl_3_): *δ* 27.4, 128.5, 131.7, 134.3, 135.4, 137.9, 138.2, 138.4, 143.1, 178.7, 185.5, 209.5. IR (KBr): ν = 1630, 1280, 1090, 1080, 810, 770 and 480 cm^−1^. Anal. Calc. for C_14_H_11_BiCl_2_O: C, 35.39; H, 2.33. Found: C, 35.45; H, 2.45%.

*(2-Acetyl-4-methylphenyl)chloro(4-methylphenyl)bismuthane* (**9a**). Yield: 87%; mp 196–198 °C; ^1^H-NMR (400 MHz, CDCl_3_): *δ* 2.25 (3H, s), 2.52 (3H, s), 2.66 (3H s), 7.30 (2H, d, *J* = 7.6 Hz), 7.40 (1H, d, *J* = 7.6 Hz), 8.03 (2H, d, *J* = 7.6 Hz), 8.13 (1H, d, *J* = 7.6 Hz), 8.13 (1H, d, *J* = 7.6 Hz), 8.85 (1H, s). ^13^C-NMR (100 MHz, CDCl_3_): *δ*21.5, 22.3, 27.3, 129.1, 132.4, 135.3, 136.4, 138.1, 139.0, 140.9, 149.8, 177.8, 185.6, 208.7. IR (KBr): ν = 1620, 1580, 1380, 800, 590 and 480 cm^−1^. Anal. Calc. for C_16_H_16_BiClO: C, 41.00; H, 3.44. Found: C, 40.78; H, 3.42%.

*(2-Acetyl-4-isopropylphenyl)chloro(4-methylphenyl)bismuthane* (**9b**). Yield: 93%; mp 198–200 °C; ^1^H-NMR (400 MHz, CDCl_3_): *δ* 1.33 (6H, d, *J* = 7.2 Hz), 2.28 (3H, s), 2.65 (3H, s), 3.13 (1H, sep, *J* = 7.2 Hz), 7.33 (2H, d, *J* = 7.6 Hz), 7.45 (1H, dd, *J* = 1.6, 8.0 Hz), 8.04 (2H, d, *J* = 7.6 Hz), 8.18 (1H, d, *J* = 8.0 Hz), 8.92 (1H, d, *J* = 1.6 Hz). ^13^C-NMR (100 MHz, CDCl_3_): *δ* 21.5, 23.5, 23.7, 27.3, 34.9, 126.2, 132.4, 135.6, 136.4, 136.8, 138.1, 141.3, 159.9, 177.8, 185.7, 208.6. IR (KBr): ν = 1620, 1580, 1360, 1280, 1060, 830, 790 and 480 cm^−1^. Anal. Calc. for C_18_H_20_BiClO: C, 43.52; H, 4.06. Found: C, 43.48; H, 4.12%.

*(2-Acetyl-4-methoxyphenyl)chloro(4-methylphenyl)bismuthane* (**9c**). Yield: 82%; mp 178–180 °C; ^1^H-NMR (400 MHz, CDCl_3_): *δ* 2.25 (3H, s), 2.61 (3H, s), 4.01 (3H, s), 7.00 (1H, dd, *J* = 2.4, 8.8 Hz), 7.34 (2H, d, *J* = 7.6 Hz), 8.04 (2H, d, *J* = 7.6 Hz), 8.16 (1H, d, *J* = 8.8 Hz), 8.70 (1H, d, *J* = 2.4 Hz). ^13^C-NMR (100 MHz, CDCl_3_): *δ*21.5, 27.1, 55.8, 115.2, 122.6, 132.5, 136.4 (2C), 137.5, 138.2, 168.8, 178.1, 189.3, 207.0. IR (KBr): ν = 1610, 1580, 1540, 1360, 1300, 1270, 1230, 1020, 820, 800, 600 and 480 cm^−1^. Anal. Calc. for C_16_H_16_BiClO_2_: C, 39.65; H, 3.33. Found: C, 39.57; H, 3.73%.

### 3.5. Synthesis of ***6*** and ***7***

A typical example is exemplified by the synthesis of **6b** and **7b**: Saturated aqueous NaBr (3 mL) was added dropwise to a stirred solution of **5b** (227 mg, 0.5 mmol) in dichloromethane (5 mL). After 15 min, the organic layer was extracted with ethyl acetate (10 mL × 3). The combined extracts were concentrated to leave an oily residue, which was crystallized from MeOH to give **6b** in 83% yield. Saturated aqueous NaI (3 mL) was added dropwise to a stirred solution of **5b** (227 mg, 0.5 mmol) in dichloromethane (5 mL). After 15 min, the organic layer was extracted with ethyl acetate (10 mL × 3). The combined extracts were concentrated to leave an oily residue, which was crystallized from MeOH to give **7b** in 89% yield.

*(2-Acetylphenyl)bromo(4-methoxylphenyl)bismuthane* (**6a**). Yield: 85%; mp 153–155 °C; ^1^H-NMR (400 MHz, CDCl_3_): *δ* 2.69 (3H, s), 3.73 (3H, s), 6.98 (2H, d, *J* = 8.4 Hz), 7.66 (1H, dt, *J* = 1.2, 7.8 Hz), 7.97 (1H, dt, *J* = 1.2, 7.6 Hz), 8.02 (2H, d, *J* = 8.4 Hz), 8.25 (1H, d, *J* = 7.6 Hz), 9.21 (1H, d, *J* = 7.2 Hz). ^13^C-NMR (100 MHz, CDCl_3_): *δ* 27.4, 55.1, 117.5, 128.4, 135.1, 138.5, 138.7, 140.7, 143.1, 159.4, 169.0, 180.7, 208.7. IR (KBr): ν = 1625, 1550, 1300, 1280, 800, 765, 615 and 480 cm^−1^. Anal. Calc. for C_15_H_14_BiBrO_2_: C, 34.97; H, 2.74. Found: C, 34.70; H, 2.70%.

*(2-Acetylphenyl)bromo(4-methylphenyl)bismuthane* (**6b**). Yield: 83%; mp 174–176 °C; ^1^H-NMR (400 MHz, CDCl_3_): *δ* 2.05 (3H, s), 2.69 (3H, s), 7.31 (2H, d, *J* = 7.9 Hz), 7.65 (1H, t, *J* = 1.1, 7.5 Hz), 7.97 (1H, t, *J* = 1.1, 7.4 Hz), 8.05 (2H, d, *J* = 7.9 Hz), 8.24 (1H, d, *J* = 7.3 Hz), 9.21 (1H, d, *J* = 7.3 Hz). ^13^C-NMR (100 MHz, CDCl_3_): *δ* 21.5, 27.4, 128.3, 132.5, 135.1, 137.1, 138.2, 138.5, 140.7, 143.1, 174.2, 175.8, 208.7. IR (KBr): ν = 1625, 1550, 1300, 1280, 800, 770, 620 and 480 cm^−1^. Anal. Calc. for C_13_H_10_BiClO_2_S: C, 36.09; H, 2.83. Found: C, 36.20; H, 2.80%.

*(2-Acetylphenyl)bromo(phenyl)bismuthane* (**6c**). Yield: 87%; mp 146–148 °C; ^1^H-NMR (400 MHz, CDCl_3_): *δ*2.70 (3H, s), 7.25 (1H, t, *J* = 7.3 Hz), 7.50 (2H, t, *J* = 7.3 Hz), 7.67 (1H, t, *J* = 7.3 Hz), 7.98 (1H, t, *J* = 7.3 Hz), 8.25 (1H, d, *J* = 7.3 Hz), 9.21 (1H, d, *J* = 7.3 Hz). ^13^C-NMR (100 MHz, CDCl_3_): *δ* 27.4, 128.2, 128.4, 131.7, 135.1, 137.1, 138.6, 140.7, 143.1, 177.3, 180.8, 208.8. IR (KBr): ν = 1620, 1280, 770 and 730 cm^−1^. Anal. Calc. for C_14_H_12_BiBrO: C, 34.66; H, 2.48. Found: C, 34.80; H, 2.40%.

*(2-Acetylphenyl)bromo(4-fluorophenyl)bismuthane* (**6d**). Yield: 83%; mp 132–134 °C; ^1^H-NMR (400 MHz, CDCl_3_): *δ* 2.70, (3H, s), 7.09–7.13 (2H, m), 7.68 (1H, t, *J* = 7.2 Hz), 7.99 (1H, t, *J* = 7.6 Hz), 8.10–8.13 (2H, m), 8.26 (1H, *J* = 7.6 Hz), 9.20 (1H, d, *J* = 7.2 Hz). ^13^C-NMR (100 MHz, CDCl_3_): *δ*27.4, 118.9 (d, *J*_CF_ = 21 Hz), 128.6, 135.1, 138.7, 139.2 (d, *J*_CF_ = 7.0 Hz), 140.6, 143.1, 162.4 (d, *J*_CF_ = 246 Hz), 172.0, 180.5, 208.9. IR (KBr): ν = 1630, 1220, 1160, 810, 610 and 500 cm^−1^. Anal. Calc. for C_14_H_11_BiBrFO: C, 33.42; H, 2.20. Found: C, 33.55; H, 2.24%.

*(2-Acetylphenyl)bromo(4-chlorophenyl)bismuthane* (**6e**). Yield: 86%; mp 157–159 °C; ^1^H-NMR (400 MHz, CDCl_3_): *δ* 2.70 (3H, s), 7.41 (2H, d, *J* = 8.4 Hz), 7.68 (1H, t, *J* = 7.6 Hz), 7.99 (1H, t, *J* = 8.0 Hz), 8.10 (2H, d, *J* = 8.4 Hz), 8.25 (1H, d, *J* = 7.6 Hz), 9.20 (1H, d, *J* = 7.6 Hz). ^13^C-NMR (100 MHz, CDCl_3_): *δ* 27.4, 128.6, 131.7, 134.3, 135.2, 138.6, 138.7, 140.6, 143.1, 174.9, 180.4, 209.0. IR (KBr): ν = 1630, 1280, 1090, 1000, 800 and 770 cm^−1^. Anal. Calc. for C_14_H_11_BiBrClO: C, 32.36; H, 2.13. Found: C, 32.70; H, 2.10%.

*(2-Acetylphenyl)bromo(2,4,6-trimethylphenyl)bismuthane* (**6f**). Yield: 86%; mp 181–183 °C; ^1^H-NMR (400 MHz, CDCl_3_): *δ* 2.19 (3H, s), 2.45 (6H, s), 2.63 (3H, s), 7.12 (2H, s), 7.64 (1H, dt, *J* = 1.2, 7.6 Hz), 7.94 (1H, dt, *J* = 0.8, 7.2 Hz), 8.21 (1H, d, *J* = 7.2 Hz), 9.35 (1H, d, *J* = 7.6 Hz). ^13^C-NMR (100 MHz, CDCl_3_): *δ* 21.2, 26.8, 27.3, 129.1, 131.2, 134.7, 137.6, 138.6, 142.2, 144.0, 146.6, 178.9, 179.3, 207.7. IR (KBr): ν = 1630, 1280, 1260 and 760 cm^−1^. Anal. Calc. for C_17_H_18_BiBrO: C, 38.73; H, 3.44. Found: C, 38.48; H, 3.55%.

*(2-Acetylphenyl)iodo(4-methoxylphenyl)bismuthane* (**7a**). Yield: 83%; mp 145–147 °C; ^1^H-NMR (400 MHz, CDCl_3_): *δ* 2.69 (3H, s), 3.73 (3H, s), 6.93 (2H, d, *J* = 8.4 Hz), 7.71 (1H, t, *J* = 7.6 Hz), 7.87 (1H, t, *J* = 7.2 Hz), 8.07 (2H, d, *J* = 8.4 Hz), 8.23 (1H, d, *J* = 7.6 Hz), 9.42 (1H, d, *J* = 7.2 Hz). ^13^C-NMR (100 MHz, CDCl_3_): *δ* 27.1, 55.0, 117.4, 128.5, 134.5, 139.0, 139.9, 143.1, 145.6, 159.4, 207.5; *ipso* carbon signals were too weak to be observed. IR (KBr): ν = 1630, 1580, 1490, 1290, 1250, 1180, 810, 760 and 510 cm^−1^. Anal. Calc. for C_14_H_13_BiClNO_2_S: C, 32.05; H, 2.51. Found: C, 32.29; H, 2.60%.

*(2-Acetylphenyl)iodo(4-methylphenyl)bismuthane* (**7b**). Yield: 89%; mp 148–150 °C; ^1^H-NMR (400 MHz, CDCl_3_): *δ*2.26 (3H, s), 2.69 (3H, s), 7.25 (2H, d, *J* = 8.0 Hz), 7.71 (1H, dt, *J* = 1.2, 7.6 Hz), 7.86 (1H, dt, *J* = 1.6, 7.9 Hz), 8.07 (2H, d, *J* = 8.0 Hz), 8.22 (1H, dd, *J* = 1.2, 8.0 Hz), 9.42 (1H, dd, *J* = 1.2, 7.2 Hz). ^13^C-NMR (100 MHz, CDCl_3_): *δ* 21.6, 27.1, 128.5, 132.4, 134.5, 138.0, 138.2, 139.0, 143.1, 145.6, 166.7, 172.1, 207.5. IR (KBr): ν = 1630, 1290, 800, 770 and 480 cm^−1^. Anal. Calc. for C_15_H_14_BiIO: C, 32.98; H, 2.59. Found: C, 33.00; H, 2.60%.

*(2-Acetylphenyl)iodo(phenyl)bismuthane* (**7c**). Yield: 88%; mp 111–113 °C; ^1^H-NMR (400 MHz, CDCl_3_): *δ* 2.69 (3H, s), 7.25 (1H, dt, *J* = 0.8 Hz, 6.4 Hz), 7.37 (2H, dt, *J* = 1.6, 7.6 Hz), 7.89 (1H, dt, *J* = 1.2, 7.6 Hz), 8.20 (2H, dd, *J* = 1.2, 7.6 Hz), 8.23 (1H, dd, *J* = 1.2, 8.0 Hz), 9.43 (1H, dd, *J* = 0.8, 7.6 Hz). ^13^C-NMR (100 MHz, CDCl_3_): *δ* 27.0, 128.0, 128.6, 131.5, 134.5, 138.2, 139.0, 143.1, 145.5, 169.8, 172.1, 207.6. IR (KBr): ν = 1630, 1430, 1280, 770, 730, 690, 610 and 460 cm^−1^. Anal. Calc. for C_14_H_12_BiIO: C, 31.60; H, 2.27. Found: C, 31.78; H, 2.60%.

*(2-Acetylphenyl)iodo(4-fluorophenyl)bismuthane* (**7d**). Yield: 76%; mp 123–125 °C; ^1^H-NMR (400 MHz, CDCl_3_): *δ* 2.70 (3H, s), 7.03-7.08 (2H, m), 7.73 (1H, dt, *J* = 0.8, 7.2 Hz), 7.90 (1H, dt, *J* = 0.8, 7.2 Hz), 8.11-8.16 (2H, m), 9.42 (1H, dd, *J* = 1.2, 7.6 Hz). ^13^C-NMR (100 MHz, CDCl_3_): *δ* 27.1, 118.8 (d, *J*_CF_ = 20 Hz), 128.7, 134.6, 139.1, 140.4 (d, *J*_CF_ = 8.0 Hz), 143.0, 145.5, 162.4 (d, *J*_CF_ = 246 Hz), 164.2, 171.7, 207.7. IR (KBr): ν = 1630, 1220, 1160, 810, 760, 610 and 500 cm^−1^. Anal. Calc. for C_14_H_11_BiFIO: C, 30.57; H, 2.02. Found: C, 30.52; H, 1.99%.

*(2-Acetylphenyl)iodo(4-chlorophenyl)bismuthane* (**7e**). Yield: 79%; mp 153–155 °C; ^1^H-NMR (400 MHz, CDCl_3_): *δ* 2.70 (3H, s), 7.43 (2H, dd, *J* = 1.6, 8.4 Hz), 7.73 (1H, dt, *J* = 1.2, 7.6 Hz), 7.90 (1H, dt, *J* = 1.2, 7.2 Hz), 8.11 (2H, dd, *J* = 1.6, 8.4 Hz), 8.24 (1H, dd, *J* = 0.8, 7.6 Hz), 9.41 (1H, dd, *J* = 0.8, 7.2 Hz). ^13^C-NMR (100 MHz, CDCl_3_): *δ* 27.1, 128.8, 131.6, 134.2, 134.6, 139.2, 139.8, 143.1, 145.5, 207.7; *ipso* carbon signals were too weak to be observed. IR (KBr): ν = 1630, 1470, 1280, 1090, 1000, 760, 610 and 480 cm^−1^. Anal. Calc. for C_14_H_11_BiClIO: C, 29.68; H, 1.96. Found: C, 29.72; H, 2.03%.

*(2-Acetylphenyl)iodo(2,4,6-trimethylphenyl)bismuthane* (**7f**). Yield: 86%; mp 170–172 °C; ^1^H-NMR (400 MHz, CDCl_3_): *δ* 2.20 (3H, s), 2.42 (6H, s), 2.63 (3H, s), 7.15 (2H, s), 7.67 (1H, dt, *J* = 0.8, 8.4 Hz), 7.84 (1H, dt, *J* = 1.2, 7.6 Hz), 8.19 (1H, d, *J* = 6.8 Hz), 9.52 (1H, d, *J* = 6.8 Hz). ^13^C-NMR (100 MHz, CDCl_3_): *δ* 21.2, 27.1, 27.9, 128.3, 130.7, 134.2, 138.1, 138.6, 144.0, 146.4, 146.6, 170.6, 172.8, 206.7. IR (KBr): ν = 1630, 1290, 1270, 850, 770 and 610 cm^−1^. Anal. Calc. for C_17_H_18_BiIO: C, 35.56; H, 3.16. Found: C, 35.56; H, 3.26%.

### 3.6. Qualitative Antifungal Assay

The yeast *S. cerevisiae* W303-1A (*MAT*a *ade2-1 can1-100 ura3-1 leu2-3,112 trp1-1 his3-11,15*) was used for the qualitative antifungal assay. Yeast extract-peptone-dextrose (YPD) plates contained 1% yeast extract, 2% peptone, 2% glucose and 1.2% agar. The cells were inoculated at a concentration of 1.3 × 10^4 ^cells/mL in YPD agar medium at 48 °C and YPD plates were immediately made in Petri dishes. Each compound was dissolved in dimethyl sulfoxide (DMSO) at a concentration of 30 mM and 5 μL of each solution was directly spotted on the surface of the plate. The plates were incubated for 48 h at 30 °C and antifungal activity was indicated by the presence of clear inhibition zones around the spot. The control experiment showed that DMSO did not inhibit fungal growth at all. In order to know the error on the inhibition zone, we carried out the antifungal assay of compound **C1** many times and confirmed that the error was within ± 1 mm.

### 3.7. Lipophilicity

The calculated logarithms of water-octanol partition coefficients (ClogP values) were obtained from the ClogP tool in Chem Draw Ultra 11.0 (Cambridge Soft, Cambridge, MA, USA).

### 3.8. DFT Calculation of the Dissociation and Association Energies

The geometries of the bismuth compounds, their dissociated cations and counter anions, and the corresponding MeS^−^-adduct anions in Table 2 were fully optimized in water through density functional theory (DFT) calculations within the polarizable continuum model (PCM) using the Gaussian 09 program package [40]. The hybrid B3LYP exchange-correlation functional and 6-31+G*/lanl2dz mixed basis set (lanl2dz effective core potential for bismuth and iodine and 6-31+G* basis set for the remaining atoms) were employed. All d functions in 6-31+G* are pure 5 D basis functions, which is the default form in the Gaussian 09 GenECP calculations. The endothermicity for the ionic dissociation and the exothermicity for the nucleophilic addition of MeS^−^ were calculated from the energies in water of each substrate, in which only the most stable conformer with the lowest energy was considered.

## 4. Conclusions

In conclusion, the present study provides insights that will aid the design of fungicidal hypervalent bismuthanes. For higher antifungal activity, adjusting the lipophilicity-hydrophilicity balance, modeling the three-dimensional molecular structure around the bismuth atom, and stabilizing the intermediate ate complex in Path 2 appeared to be more important than tuning the Lewis acidity at the bismuth atom. Further studies are now underway to test this hypothesis.
